# The SBP-Box Gene *VpSBP11* from Chinese Wild *Vitis* Is Involved in Floral Transition and Affects Leaf Development

**DOI:** 10.3390/ijms18071493

**Published:** 2017-07-13

**Authors:** Hongmin Hou, Xiaoxiao Yan, Ting Sha, Qin Yan, Xiping Wang

**Affiliations:** 1 State Key Laboratory of Crop Stress Biology in Arid Areas, College of Horticulture, Northwest A&F University, Yangling 712100, China; hmhou@qau.edu.cn (H.H.); xiaoxyan@nwafu.edu.cn (X.Y.); yanqin0421@gmail.com (Q.Y.); 2 Key Laboratory of Horticultural Plant Biology and Germplasm Innovation in Northwest China, Ministry of Agriculture, Yangling 712100, China; 3 College of Horticulture, Qingdao Agricultural University, Qingdao 266109, China; shating789000@gmail.com

**Keywords:** *Vitis pseudoreticulata*, *VpSBP11*, floral transition

## Abstract

Flowering occurs in angiosperms during a major developmental transition from vegetative growth to the reproductive phase. *Squamosa* promoter binding protein (SBP)-box genes have been found to play critical roles in regulating flower and fruit development, but their roles in grapevine have remained unclear. To better understand the functions of the grape SBP-box genes in both vegetative and reproductive growth phases, a full-length complementary DNA (cDNA) sequence of the putative SBP-box transcription factor gene, *VpSBP11*, was obtained from Chinese wild grapevine *Vitis pseudoreticulata* Wen Tsai Wang (W. T. Wang) clone ‘Baihe-35-1’. *VpSBP11* encoded a putative polypeptide of 170 amino acids with a highly conserved SBP-domain with two zinc-binding sites of the Cx2C-x3-H-x11-C-x6-H (C2HCH) type and a nuclear localization signal. We confirmed that the VpSBP11 protein was targeted to the nucleus and possessed transcriptional activation activity by subcellular localization and *trans*-activation assay. Over-expression of *VpSBP11* in *Arabidopsis thaliana* was shown to activate the *FUL* gene, and subsequently the *AP1* and *LFY* genes, all of which were floral meristem identity genes, and to cause earlier flowering than in wild type (WT) plants. The pattern of vegetative growth was also different between the transgenic and WT plants. For example, in the *VpSBP11* over-expressing transgenic plants, the number of rosette leaves was less than that of WT; the petiole was significantly elongated; and the rosette and cauline leaves curled upwards or downwards. These results were consistent with *VpSBP11* acting as a transcription factor during the transition from the vegetative stage to the reproductive stage.

## 1. Introduction

Flowering occurs in angiosperms during a major developmental transition from vegetative growth to the reproductive phase [1]. In *Arabidopsis thaliana*, flowering time is regulated by five converging pathways: autonomous, gibberellic acid, photoperiod, thermosensory, and vernalization [2,3,4]. The formation of normal flowers requires the signals of flowering which are integrated by floral integrator genes such as *SUPPRESSOR OF OVEREXPRESSION OF CONSTANS 1* (*SOC1*), *FLOWERING LOCUS T* (*FT*) and *FLOWERING LOCUS D* (*FD*) [5,6,7,8], and the expression of the flower-meristem-identity genes including *FRUITFULL* (*FUL*), *APETALA1* (*AP1*), *LEAFY* (*LFY*), *Squamosa*, and so on [9,10,11,12]. Although numerous studies have reported several internal and external factors, such as developmental age, the phytohormone gibberellic acid, and ambient temperature, as being involved in the regulation of flowering, much remains to be learned about the molecular mechanisms underlying the establishment of floral identity in meristems.

*Squamosa* promoter binding proteins (SBP)-box genes encode a family of transcription factors which are exclusively identified in plants [13]. The SBP-box genes all contain a highly conserved DNA-binding domain (SBP domain) which includes a putative nuclear localization signal and two zinc-binding sites [14]. SBP-box genes were first discovered in *Antirrhinum majus* and two genes named *AmSBP1* and *-2* were identified based on their ability to interact with the promoter sequence region of the floral meristem identity gene *Squamosa* [15]. Recent functional studies involving a taxonomically broad range of plant species have suggested that *SBP* plays diverse roles in plant development, including regulating developmental transitions from juvenile to adult growth and vegetative to reproductive growth [16,17,18,19,20,21,22]. Other reports have indicated that *SBP* genes regulate flower and leaf development. For example, the snapdragon gene, *AmSBP1*, as well as the *Arabidopsis thaliana Squamosa* promoter binding protein-like (SPL) genes *AtSPL3* and *AtSPL4*, bound *cis*-elements in the promoters of the floral organ identity genes *SQUA* and *APETALA1* (*AP1*) [15], and they have also been implicated in the vegetative phase change and floral induction [13,15,23]. Over-expression of the *A. thaliana* gene, *AtSPL3*, resulted in increasing expression of *AP1*, *FRUITFULL* (*FUL*), and *LEAFY* (*LFY*) used in the identification of floral induction and, as a consequence, early flowering [21]. *AtSPL9* and *AtSPL15* were active in the vegetative shoot apex and played a role in the juvenile-to-adult phase transition [18], and *AtSPL10/11/12* were involved in the development of lateral organs, shaping of cauline leaves, and in determining the number of trichomes on cauline leaves and flowers [24]. In other studies, the rice gene, *OsSPL14*, was shown to promote panicle branching in the vegetative stage and to increase yield in the reproductive stage [25], while *ZmSBP6* (*tasselsheath4*) has been found to be regulate inflorescence development in maize, and an SBP-box gene from tomato (*Solanum lycopersicum*) (*CNR*) played a role in fruit ripening [26,27]. Three homologous genes, *AtSPL13*, *TEOSINTE GLUME ARCHITECTURE 1* (*TGA1*), and *OsSPL6*, all played critical roles in the vegetative and reproductive phases: *AtSPL13* gene has been shown previously to affect the initiation of the first true leaves [28], maize *tga1* was involved in ear glume development [29], and *OsSPL16* controlled grain size, shape, and quality in rice [30]. Moreover, the petunia gene *PhSBP1* can accelerate leaf initiation rate and control the timing of developmental phase change [22], while *PaSPL3a/b/c/d* from *Platanus acerifolia* were found to induce early flowering and control vegetative to reproductive phase change [31].

Grape is cultivated worldwide and used not only as a fresh fruit but in processed food products such as juice or wine with great economic value [32]. While *SBP* genes from a range of plant species have been characterized, the functions of grape (*Vitis vinifera*) *SBP* genes, and their associated roles in regulating vegetative and reproductive growth, remained unreported. Wild Chinese grape (*V. pseudoreticulata*) is one valuable resource for future grape breeding programs; it exhibits high resistance to a variety of pathogens, and it has now become an important source for the study of more momentous genes [33]. We have identified eighteen SBP-box gene family members from *V. vinifera*, and a synteny analysis between these and the model plant *A. thaliana* homologs showed that *VvSBP11* and *AtSPL4, 5* were ortholog pairs [34]. In the present study, we report the molecular cloning and first characterization of a SBP-box gene (*VpSBP11*) from the *V. pseudoreticulata* clone ‘Baihe-35-1’. We further demonstrate that VpSBP11 protein localized to the nucleus and possessed a transcriptional activation domain, consistent with its predicted function as a transcription factor. Over-expression of *VpSBP11* in *A. thaliana* led to accelerated flowering and changes in leaf morphology and number. The results of this study provide a foundation for functional characterization of the *VpSBP11* gene and advance our understanding of the mechanism regulating flowering in grape.

## 2. Results

### 2.1. Cloning and Sequence Analysis of VpSBP11

**Figure 1:**
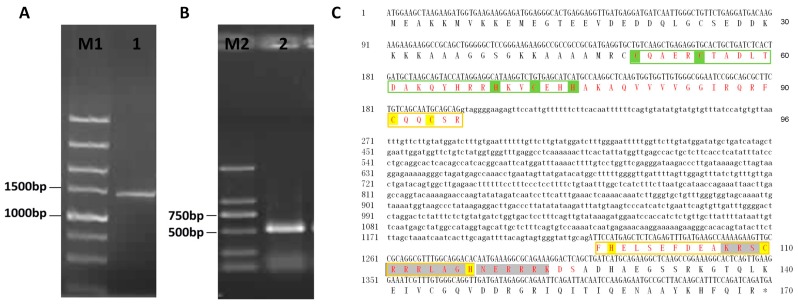
Cloning and sequence analysis of *VpSBP11* from *V*. *pesudoreticulata*. (**A**) PCR amplification of the full length *VpSBP11* DNA and (**B**) complementary DNA (cDNA) from *Vitis pseudoreticulata*. M1: DNA marker DL5000; M2: DNA marker DL2000; 1: *VpSBP11* PCR product from DNA; 2: *VpSBP11* PCR product from cDNA; (**C**) The full length DNA, cDNA nucleotide sequence, and deduced amino acid sequence of *VpSBP11* from *V*. *pesudoreticulata*. The *Squamosa* promoter binding protein (SBP) domain is shown in red and the two zinc-binding sites of the C2HCH type (zinc finger 1 and zinc finger 2) are shown in the green and yellow boxes, respectively. The conserved basic amino acids of the nuclear location signal are shaded in dark grey.

A 1440 base pair (bp) full-length *VpSBP11* DNA sequence (Figure 1A), including a 510 bp open reading frame (ORF) (Figure 1B), was amplified from genomic DNA or complementary DNA (cDNA) extracted from the leaves of *V. pseudoreticulata* W. T. Wang clone ‘Baihe-35-1’. The resulting PCR product was cloned into the pGEM-Teasy vector (Promega, Madison, WI, USA) and transformed into *Escherichia coli* strain DH5α prior to sequencing. A comparison between the genomic DNA nucleotide sequence and cDNA sequence was performed to determine the position and sequence of the introns, and it was found that the coding region of *VpSBP11* has one intron of 930 bp (Figure 1C). Moreover, the sequence of *VpSBP11* gene amplification was consistent with that of *VvSBP11* (XM_002275692.1; GSVIVT01020578001) reported in the GenBank non-redundant protein database, as well as the Grape Genome Database (12×) (http://www.genoscope.cns.fr). Previous studies have shown that *VvSBP11* had a *miR156/157* target site within its 3′ untranslated region (UTR), as did *A. thaliana AtSPL3*, *AtSPL4*, and *AtSPL*5 [34]. In addition, the *VpSBP11* contained a highly conserved SBP-domain, bearing two zinc-binding sites of the C2HCH type (zinc finger 1 and zinc finger 2), together with a nuclear localization signal (NLS) (Figure 1C).

### 2.2. Subcellular Localization and Function of VpSBP11 in Transcriptional Activation

**Figure 2:**
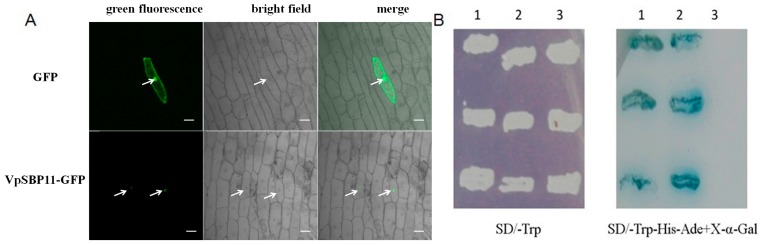
Subcellular localization and transcriptional activation function of VpSBP11 protein. (**A**) Subcellular localization of the VpSBP11-GFP (bottom row) fusion protein and GFP (top row) in onion epidermal cells. Green fluorescence of VpSBP11-GFP occurs only in the nucleus obviously while that of GFP occurs in both the nucleus and cell membrane. White arrowheads indicate the location of the nucleus in onion epidermal cell, scale bars: 50 μm; (**B**) Transcriptional activation function of *VpSBP11* in yeast. Yeast cells containing the different plasmids grown on SD/-Trp select medium (left). Yeast cells containing the different plasmids grown on SD/-Trp-His-Ade+X-α-gal selection medium (right). 1: Positive control (pGBKT7-Gal4); 2: pGBKT7-VpSBP11; 3: Negative control (pGBKT7). The experiments were repeated three times with consistent results. GFP = green fluorescent protein.

Sequence analysis of the grape SBP-box genes revealed that their deduced protein sequences contained putative NLS regions (Figure 1C). To confirm targeting of *VpSBP11* to the nucleus, the *VpSBP11* coding sequence (CDS) was translationally fused to green fluorescent protein (GFP) in the pBI221-GFP vector, and this vector, or a pBI221-GFP control vector, were transformed into onion epidermal cells using particle bombardment. The VpSBP11-GFP fusion protein was observed only in the nucleus of onion epidermal cells, whereas the GFP control protein was distributed throughout the whole cell (Figure 2A), supporting the prediction that VpSBP11 was a nuclear protein that functioned as a transcription factor.

To investigate whether VpSBP11 protein had transcriptional activation activity, the full-length *VpSBP11* CDS or the yeast *GAL4* cDNA were separately fused to the *GAL4* DNA-binding domain in the pGBKT7 vector. Yeast cells transformed with the pGBKT7 control vector only grew on Single Dropout (-Trp) (SD/-Trp) medium, while those transformed with the *VpSBP11* and *GAL4* plasmids were capable of growth on both SD/-Trp and SD/-Trp/-Ade/-His media, and exhibited blue staining in X-α-gal solution (Figure 2B), demonstrating that the *VpSBP11* protein functioned as a transcriptional activator.

### 2.3. The Effect of an VpSBP11 Transgene on Flowering Time

**Figure 3:**
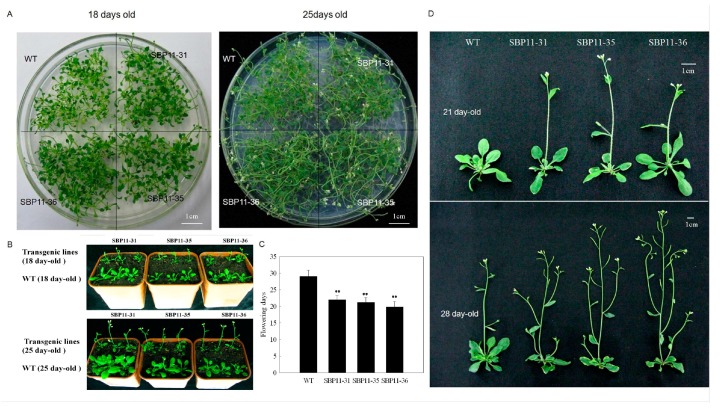
Phenotypes of *A. thaliana* wild-types (WT) and transgenic lines overexpressing *VpSBP11.* (**A**) Three transgenic lines and WT grown together in the same petri dish (18 and 25 days), scale bars: 1 cm; (**B**) Three transgenic lines and WT grown together in the same pot (18 and 25 days); (**C**) Statistics about flowering times of 100 plants of three transgenic lines and WT grown together in the same pot. Asterisks indicate statistical significance (** *p* < 0.01, one-way ANOVA); (**D**) Phenotypes of three T3 generations of transgenic plants and WT (21 and 28 days). The experiments were repeated three times with consistent results, scale bars: 1 cm.

*VpSBP11* had a predicted *miR156/157* target site within its 3′UTR, as had *AtSPL3*, *AtSPL4*, and *AtSPL*5 [34], indicating that these genes may have the same, or similar, functions. In order to investigate the role of *VpSBP11*, we made transgenic *A. thaliana* plants expressing the *VpSBP11* ORF (without the miR156 target site, which was located in the 3′UTR) under the regulation of the constitutive CaMV 35S promoter. We compared the flowering times of wild-type (WT) Columbia-0 and transgenic plants and observed that the 68 T2 transgenic lines showed earlier flowering. We selected the three lines that flowered the earliest (SBP11-31, SBP11-35, SBP11-36) for further study, and grew them in the same Petri dish as WT plants grown side by side under normal light conditions (Figure 3B). The three transgenic lines flowered 7 days earlier than the WT plants and bolting occurred after 18 days of growth in the transgenic lines, but after 25 days in the WT (Figure 3A,B). When the flowering times of 100 plants of the transgenic lines (SBP11-31, SBP11-35, SBP11-36) and WT grown in the same pot were measured, they were statistically different at 7 days (Figure 3B,C).

We observed the morphological flowering characteristics of the transgenic and WT plants grown for 21 and 28 days. The first inflorescence of 21-day-old Pro 35S: *VpSBP11* transgenic plants were beginning to bloom and the second branch was beginning to grow, while the 21-day-old WT was still in the vegetative stage (Figure 3D). After 28 days, the first branch of the transgenic plants was already in the fructification stage and the second branch was beginning to bloom, while a third and fourth branch were starting to grow. At this time, only the first inflorescence of the WT was beginning to bloom (Figure 3D).

To better understand the histological features of early flowering, we also observed the formation of the floral primordium for 13 days after germination using methods of scanning electron microscope (SEM) analysis and paraffin sectioning (Figure S1). There were no differences at 9 days between *VpSBP11* transgenic lines and WT. The transgenic lines (SBP11-31, SBP11-35, SBP11-36) formed flower primordia 11 days after germination, while the WT formed flower primordia 13 days after germination (Figure S1). Taken together, these results indicated that over-expression of *VpSBP11* without the microRNA target site in the 3′UTR promoted early flowering compared to the WT.

### 2.4. Over-Expression of VpSBP11 Upregulated LFY, FUL and AP1

**Figure 4:**
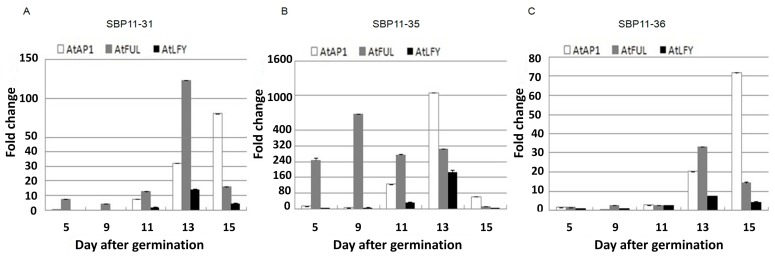
The meristem identity switch genes (*AtAP1*, *AtFUL*, and *AtLFY*) expression in *VpSBP11* transgenic lines. (**A**) SBP11-31; (**B**) SBP11-35 and (**C**) SBP11-36 compared to wild-type plants. *AtActin1* was used as an internal control for qRT-PCR and fold changes were used to indicate expression levels in leaves compared to WT controls. Mean values and SDs were obtained from three biological experiments with consistent results.

We reasoned that the precocious meristem identity transition in 35S: *VvSBP11* could be due to upregulation of genes that were upstream regulators of floral meristem identity (*LFY*, *FUL* and *AP1*). To test this possibility, we examined their expression in the transgenic lines (SBP11-31, SBP11-35, SBP11-36) and in WT plants before and after the onset of the reproductive stage (5, 9, 11, 13 and 15 days after germination) by using quantitative real-time PCR (qRT-PCR) analysis. We observed that the three floral meristem identity genes were expressed at higher levels in the transgenic lines than in WT, although to differing degrees (Figure 4). In the transgenic SBP11-35 line 9 days after germination, *FUL* was expressed >600-fold higher than in WT (Figure 4B). Similarly, the expression of *FUL* was up-regulated >100-fold and >30-fold in the SBP11-31 and SBP11-36 transgenic lines, respectively, 13 days after germination. As the plants grew, *FUL* expression began to rapidly decline, while *AP1* and *LFY* expression showed a sharp increase (Figure 4A,C). In the SBP11-35 transgenic lines, *AP1* expression was up-regulated >1000-fold at 13 days compared to WT (Figure 4B), while it reached maximum expression at day 15 in line SBP11-31 and SBP11-36, which was >70-fold greater than in WT (Figure 4A,C). Compared to the other two genes, *LFY* was not as obviously up-regulated; however, its expression was also greater in the transgenic lines than in WT: >10, >160-fold and >7-fold higher in SBP11-31, SBP11-35, and SBP11-36, respectively (Figure 4). The data indicated that over-expression of *VpSBP11* first activated *FUL* and, subsequently, *AP1* and *LFY*.

### 2.5. Regulation of the Vegetative Phase Change

**Figure 5:**
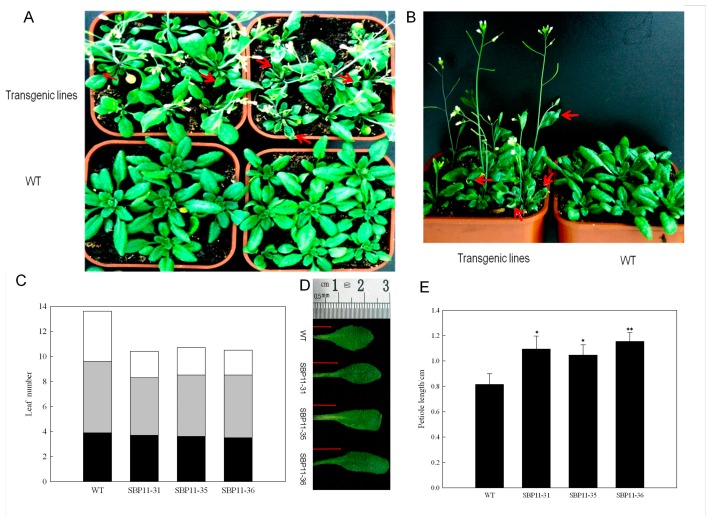
Phenotype of *VpSBP11* transgenic plants. (**A**) Wild-type (WT) plant compared with *VpSBP11* transgenic lines after 21 days of growth under long-day conditions. The transgenic plants flowered earlier and formed fewer leaves; (**B**) The rosette and cauline leaves of the *VpSBP11* transgenic lines were strongly curled. Red arrowheads in (**A**,**B**) indicate the strongly curled leaves of the transgenic plants; (**C**) The number of leaves without abaxial trichomes (black), with abaxial trichomes (gray), and with cauline leaves (white) of three *VpSBP11* transgenic lines (SBP11-31, SBP11-35, and SBP11-36) (*n* > 30); (**D**) Representative blade petiole lengths of rosette leaves of the *VpSBP11* transgenic lines. The red lines visually indicate the length of the representative blade petiole; (**E**) Petiole lengths of rosette leaves of the genotypes illustrated in (**A**) (21 days, *n* = 50 for each genotype, * *p* < 0.05, ** *p* < 0.01, one-way ANOVA). The experiments were repeated three times with consistent results.

Many molecular genetic studies have shown that *VpSBP11* homologs from other species also regulate vegetative phase change in addition to floral induction [13,17,25]. To further elucidate the function of *VpSBP11*, we compared the leaf developmental patterns in WT and 35S: SBP11 transgenic plants. Consistent with previous observations, an analysis of leaf morphology, leaf number, and abaxial trichomes at different developmental phases revealed that the pattern of vegetative phase changes was different (Figure 5). For example, in the 35S: *VpSBP11* transgenic plants, unlike in the control plants, the lamina of later rosette and cauline leaves curled upwards or downwards (Figure 5A,B). Leaves of *A. thaliana* were artificially divided into three categories: leaves without abaxial trichomes, leaves with abaxial trichomes, and cauline leaves. Here, there were obviously fewer leaves with abaxial trichomes of transgenic plants than in WT, and two times fewer cauline leaves than in WT (Figure 5A,C), and the petiole length of rosette leaves of transgenic plants was significantly (1.28–1.42-fold) longer than that of WT (Figure 5D,E).

### 2.6. Spatial VpSBP11 Expression Pattern

**Figure 6:**
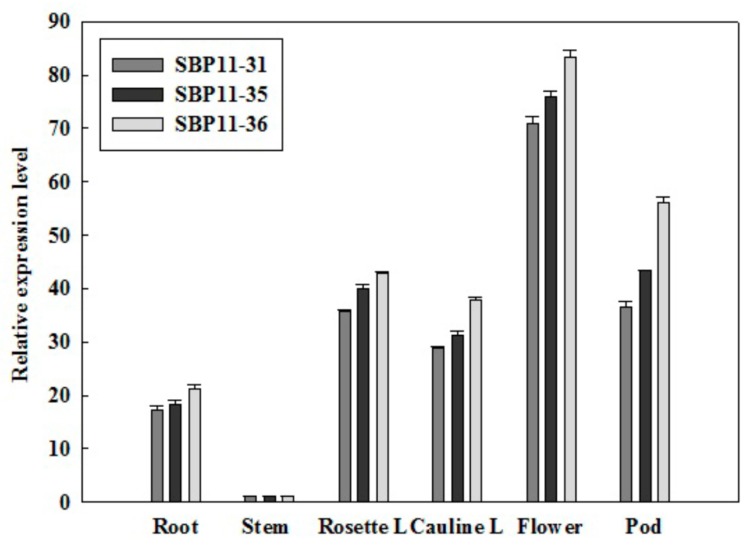
Spatial expression pattern of *VpSBP11*. The relative expression levels of *VpSBP11* were measured in different organs of WT and three transgenic lines (SBP11-31, SBP11-35, and SBP11-36): Root, Stem, Rosette Leaf (Rosette L), Cauline Leaf (Cauline L), Flower, and Pod. Expression in the stem was the lowest and was selected as the base value of comparison. *AtActin1* was used as an internal control for qRT-PCR. Mean values and SDs were obtained from three biological experiments with consistent results.

Since the expression of *SBP* genes was sensitive in the vegetative to reproductive stage transition, subsequent analyses focused on the spatial expression pattern of *VpSBP11*. Various organs of WT and transgenic plants were collected (Figure 6) to compare the difference of expression patterns of *VpSBP11* during the course of plant growth and development. In general, expression of *VpSBP11* was higher in the reproductive than in the vegetative organs. For example, it was highest in the flower of transgenic lines but lowest in the stem, which was selected as the base value of comparison between organs. *VpSBP11* expression in the root (17–22-fold), rosette leaves (35–43-fold), and cauline leaves (28–38-fold) were shown in the three transgenic lines (SBP11-31, SBP11-35, SBP11-36) compared to that in the stem. Moreover, *VpSBP11* expression was higher in the flowers (70–84-fold) and pods (36–57-fold) in the three transgenic lines compared to that in the stem. These results suggest that the expression of *VpSBP11* is regulated during the transition from the vegetative stage to the reproductive stage.

## 3. Discussion

### 3.1. VpSBP11 Sequence Analysis

The SBP-box gene family encodes DNA binding proteins that are putative plant-specific transcription factors and are present in green plants from single-celled algae to the most highly developed plants. The SBP-domain is approximately 74 amino acid residues, and harbors a highly conserved DNA-binding domain (the *Squamosa* promoter binding protein [SBP] domain), which features a zinc finger motif with two zinc-binding sites [14]. The *VpSBP11* amino acid sequence contains all the features typical of SBP-box proteins including the SBP-box and a putative nuclear localization signal (Figure 2). We further confirmed that *VpSBP11* is targeted to the nucleus and possesses transcriptional activation activity.

### 3.2. The Grape VpSBP11 Gene Regulated Flowering Time and Affected Leaf Development

Previous studies have shown that, as was that case with *AtSPL3*, *AtSPL4*, and *AtSPL*5 from *A. thaliana*, *VvSBP11* belonged to the Group 4 clade [34]. Moreover, expression of *VvSBP11* was reported to be higher in the inflorescence and young fruit stages, then gradually decreased or was absent at the veraison stage [34]. The sequence of the *VpSBP11* gene is the same as *VvSBP11* (XM_002275692.1; GSVIVT01020578001) reported in the GenBank non-redundant protein database, as well as the Grape Genome Database (12×) (http://www.genoscope.cns.fr). We, therefore, speculated that *VpSBP11* might have a role in flower and fruit development. Here, we found that *VpSBP11* over-expression in *A. thaliana* led to higher *VpSBP11* expression levels in reproductive than in vegetative organs, especially in flowers. This result was consistent with previous studies showing that the *VpSBP11* homologs, *AmSBP1* from snapdragon and *AtSPL3*/*4*/*5* from *A. thaliana*, bind to a *cis*-elements in the promoter of the floral organ identity genes *SQUA* and *AP1* [15] and were all involved in the vegetative phase change and floral induction [13,15,23].

Recently, SBP-box transcription factors have been implicated in the regulation of multiple developmental transitions in *A. thaliana* and other plant species [13,17,18,27,35,36]. To further understand the correlation between developmental transitions and *VpSBP11* expression, we observed the formation of the floral primordium and saw that the transgenic lines (SBP11-31, SBP11-35, SBP11-36) formed flower primordia 11 days after germination, which was 2 days earlier than that of WT. In addition, the flowering time of the transgenic lines was 7 days earlier than that of the WT (Figure 2). We showed here that the *AtSPL3* homolog, *VpSBP11*, may be able to regulate one of the developmental transitions when over-expressed in *A. thaliana*, suggesting a direct molecular link between SBP transcription factors and one of the developmental transitions that they regulate. In addition to accelerating flower initiation, the functions of the *SBP* homologs in the vegetative phase have been described in several plant species [18,19,22,24,25]. We showed here that the over-expression of *VpSBP11* led to changes in leaf morphology and numbers (Figure 5).

### 3.3. VpSBP11 Regulated Expression of Floral Meristem Identity Genes

The floral meristem identity genes, *LFY*, *AP1*, and *FUL*, were master regulators that controlled the correct timing of flower transition. Studies in *A. thaliana* have shown that their expression increased immediately prior to the meristem identity transition, with *LFY* and *FUL* activated very early and *AP1* upregulation occurring later [37]. Previous studies have also suggested that several *SPL* transcription factors were able to directly activate the expression of *LFY*, *FUL*, and *AP1* to promote floral meristem identity during the floral transition [21].

For example, expression of *AtSPL*3, which binds to a conserved motif in the promoter of the *AP1* ortholog *Squamosa*, preceded the activation of *AP1* at the shoot apex [13]. Furthermore, *LFY* introns were known to be critical for proper expression of *LFY* in monocots [38,39], and *AtSPL3* bound strongly in vivo to a region of *LFY* that contained three consensus SBP binding sites [21]. The MADS-box transcription factor, *FUL*, played a role in both the reproductive transition and the meristem identity transition together with other MADS-box transcription factors [40]. More recently, *AtSPL3* was identified as a direct upstream activator of *FUL*. It was shown to control the developmental timing at multiple stages in the plant life cycle and to be an important target for *SPL* transcription factors [21]. Consistent with this, we found that the three floral meristem identity genes were all activated, although to different degrees, in the transgenic *A. thaliana* lines (SBP11-31, SBP11-35, and SBP11-36). We concluded that over-expressing *VpSBP11* first activated the *FUL* gene and, subsequently, the *AP1* and *LFY* genes (Figure 4). We observed that most of the transgenic lines formed floral primordia 11 days after germination, which was 2 days earlier than that in WT (Figure S1). While the expression of *FUL*, *AP1*, and *LFY* was activated in the transgenic lines before the flower primordia appeared (5 days after germination), their expression peaked after 13 days (Figure 4). These results, combined with those of previous studies, support the idea that the *AtSPL3* homolog, *VpSBP11*, regulates the developmental transitions when over-expressed in *A. thaliana* by activating the expression of the floral meristem identity genes, *LFY*, *FUL*, and *AP1*.

## 4. Materials and Methods

### 4.1. Plant Material and Treatments

Chinese wild grapevine (*V. pseudoreticulata* ‘Baihe-35-1’), used for cloning of *VpSBP11*, was grown in the grape repository of Northwest A&F University, Yangling, China (34°20′ N, 108°24′ E). *A. thaliana* plants (transgenic lines and WT Columbia-0) were grown at 22 °C, 70% relative humidity, and in long day (8 h dark, 16 h light) conditions. All experiments were repeated in triplicate, and all samples were immediately frozen in liquid nitrogen and stored at −80 °C until further use.

### 4.2. Cloning of VpSBP11 and Sequence Analysis

Total RNA was extracted from *V. pseudoreticulata* ‘Baihe-35-1’ leaves, using a previously described protocol [41], then was treated with DNase I to remove DNA contamination before cDNA synthesis. The cDNA was synthesized from 1.0 µg total RNA using 500 ng of random hexamers and the M-MLV reverse transcriptase (Promega, Beijing, China). A pair of gene-specific primers (*VpSBP11-*F1 and *VpSBP11-*R1) (Table 1) were used to amplify the predicted *VpSBP11* ORF from the cDNA template with *Taq* DNA polymerase (TaKaRa Biotechnology, Dalian, China) and the following cycling program: 94 °C for 3 min, 35 cycles at 94 °C for 30 s, 58 °C for 30 s, 72 °C for 2 min, and extension at 72 °C for 10 min. The amplified products were cloned into the pGEM-Teasy vector (Promega) to generate pGEM-Teasy-VpSBP11 and transformed into the *E.coli* strain DH5α. Positive clones, were sequenced at TaKaRa Biotechnology. The conserved sequences were analyzed using Conserved Domains (http://www.ncbi.nlm.nih.gov/Structure/cdd/wrpsb.cgi). The subcellular localization of *VpSBP11* was predicted using the Center for Biological Sequence analysis software (http://genome.cbs.dtu.dk/services/TargetP; http://genome.cbs.dtu. dk/services/SignalP/).

### 4.3. Quantitative Real-Time RT-PCR Analysis

Total *A. thaliana* RNA was extracted from the entire plant and different organs (root/stem/rosette leaf/cauline leaf/flower/fruit) of WT and three transgenic lines (SBP11-31, SBP11-35, and SBP11-36) using the E.Z.N.A.^®^ Plant RNA Kit (Omega Bio-tek, Norcross, GA, USA, R6827-01). First-strand cDNA for expression analysis was synthesized from 1 µg of DNase-treated total RNA using PrimeScript™ RTase (TaKaRa Biotechnology). *Atactin1* (At2g37620) was amplified for use as an internal control. The gene specific primer pairs used for qRT-PCR were as follows: *VpSBP11-*F2 and R2, *AtLFY-*F and R (At5g61850), *AtFUL-*F and R (At5g60910), *AtAP1-*F and R (At1g69120), as well as *AtActin1-*F and R (At2g37620) (Table 1). Quantitative RT-PCR was conducted using SYBR green (Takara Biotechnology) with an IQ5 real time PCR machine (Bio-Rad, Hercules, CA, USA). The 25 µL PCR reaction contained 12.5 µL of SYBR^®^ Premix Ex Taq TM II (2×), 1 µL of PCR forward primer (10 µm), 1 µL of PCR reverse primer (10 µm), 2 µL of 10× diluted cDNA, and 8.5 µL of ddH_2_O. Cycling parameters were 95 °C for 30 s, 40 cycles of 95 °C for 5 s, and 60 °C for 30 s. For dissociation curve analysis, a program including 95 °C for 15 s, followed by a constant increase from 60 to 95 °C was included after the PCR cycles. Each reaction was done in triplicate and was analyzed using the protocol described by Gao et al. [42].

### 4.4. Subcellular Localization

The *VpSBP11* CDS with *Xba*I and *Kpn*I sites, but without the termination codon, was amplified using *VpSBP11-*F3 and R3 (Table 1) from the pGEM-Teasy-*VpSBP11* plasmid template with *Taq* DNA polymerase (TaKaRa Biotechnology). The following cycling program was followed: 94 °C for 3 min, 35 cycles at 94 °C for 30 s, 58 °C for 30 s, 72 °C for 2 min, and extension at 72 °C for 10 min. The amplified products were cloned into the pGEM-Teasy vector (Promega) and transformed into the *E. coli* strain DH5α. It was then inserted immediately upstream of, and in frame with, the green fluorescent protein (GFP) coding sequence in the pBI221-GFP vector (Clontech Laboratories, Inc., Palo Alto, CA, USA), which had been digested with *Xba*I and *Kpn*I, to generate pBI221-VpSBP11-GFP. Both the SBP11-containing vector and a background control vector with no insert were delivered into onion epidermal cells using a PDS-1000/He gene gun (Bio-Rad Laboratories Inc., Hercules, CA, USA) at 1100 psi as previously described [43], and then the cells were cultured in MS media in the dark at 22 °C for 18 h. Following cultivation, GFP accumulation was visualized using a Zeiss confocal microscope (LSM510; Carl Zeiss, Thornwood, NY, USA) with an excitation wavelength of 480 ± 20 nm and an emission wavelength of 510 ± 20 nm.

### 4.5. Trans-Activation Assay

The coding regions of yeast *GAL4* and grape *VpSBP11* were separately ligated into the *Nco*I*/Bam*HI and *Xma*I*/Bam*HI sites of the GAL4 DNA-binding domain of the pGBKT7 vector (Clontech Laboratories, Inc.) to produce plasmid pGBKT7-Gal4 (positive control) and pGBKT7-*VpSBP11*, using DNA fragments amplified with the gene-specific primers Gal4-F and R and *VpSBP11* F4 and R4 (Table 1). The *trans*-activation assay used a yeast assay system as described previously [44]. The resulting plasmids, pGBKT7-Gal4 and pGBKT7-VpSBP11, as well as the empty vector pGBKT7 (negative control) were transformed into yeast (*Saccharomyces cerevisiae*) AH109 cells, which were then streaked on SD/-Trp and SD/-Trp/-Ade/-His/X-α-Gal plates to observe yeast growth at 30 °C for 3–4 days.

### 4.6. Generation of Transgenic A. thaliana Plants Over-Expressing the Grapevine VpSBP11 Gene

The coding regions of *VpSBP11* were amplified from the pGEM-Teasy-*VpSBP11* plasmid template with *Taq* DNA polymerase (TaKaRa Biotechnology) using the gene-specific primers *VpSBP11-*F5 and R5 (Table 1), then inserted immediately into the downstream of the CaMV 35S promoter in the plant overexpression vector pCambia2300 (Clontech Laboratories, Inc.) to produce the plasmid pCambia2300-35S-VpSBP11. This was then introduced into *Agrobacterium tumefaciens* strain EHA105, which was in turn used to transform *A. thaliana* via the floral dip method [45]. Transgenic seeds (T1) were selected on MS agar medium supplemented with 60 mg·L^−1^ kanamycin. The *Arabidopsis* flowering times, defined as the opening time of the first flower bud, of wild-type and transgenic plants were noted. Of 68 T2 transgenic lines showing early flowering, the three T3 homozygous lines with the most obvious phenotypes (SBP11-31, SBP11-35, SBP11-36) were selected for further study. The petiole lengths were measured with a ruler using 50 rosette leaves with the most primitive and complete petioles sheared by a thin blade. In addition, wild-type and transgenic *Arabidopsis* plants in bloom were photographed in the petri dish, pots, and in the form of individual plants, which were used to show different phenotypes.

### 4.7. Observation of Morphology of VpSBP11 Over-Expression Strains and Wild Type

The whole plant of wild-type and transgenic lines (SBP11-31, SBP11-35, SBP11-36) were collected at 7, 9, 11 and 13 days after germination to analyze flower primordia transformation by adopting scanning electron microscopy (SEM) [46] and mature paraffin sectioning [47]. The SEM method was adopted as follows: all samples were fixed and vacuumed in 4% glutaraldehyde (20% 0.1 mol·L^−1^ NaH_2_PO_4_, 30% 0.1 mol·L^−1^ Na_2_HPO_4_, 4% glutaraldehyde and 20 g·L^−1^ activated charcoal, pH = 7.4). Samples were dehydrated through an ethanol series (30%, 50%, 70%, 90%, 95% and 100%) and stored in 70% ethanol at 4 °C until further use. After infiltrated with isoamyl acetate, samples were dried by carbon dioxide critical point drying method and sprayed with gold on the metal platform. Samples were observed using a scanning electron microscope (JSM-6360LV, Tokyo, Japan). In addition, following fixation in FAA fluid (5% formalin, 45% absolute ethanol and 5% glacial acetic acid) for 20–24 h at room temperature, all samples were dehydrated through an ethanol series (50%, 70%, 80%, 90%, 95% and 100%) and stored in 70% ethanol at 4 °C until further use. Samples stored at 4 °C were first infiltrated with xylene:paraffin (1:1) (Taiva, Hubei, China) at 38 °C for 24–48 h and then embedded in pure paraffin at 68 °C for one week. Section 8–10 µm in thickness with formed samples were transferred onto poly-l-Lys-coated glass slides (WHB, Shanghai, China), deparaffinized with xylene, and re-hydrated through an ethanol series (100%, 95%, 85%, 70%, 60%, and 30%). The resulting sections were stained with Ehrlich′s haematoxylin (Saichi, Shanghai, China) for 30 min at room temperature, dehydrated with an ethanol series, infiltrated with xylene, sealed with resinene (XT, Beijing, China), and finally mounted beneath a coverslip. Slides were observed using an optical microscope (OLYMPUS BH-2, Tokyo, Japan).

### 4.8. Statistical Analysis

Data are presented as means and standard errors using SigmaPlot 10.0 (Systat Software, Inc., Chicago, IL, USA). One-way ANOVA analysis was performed using the SPSS Statistics 17.0 software (IBM China Company Ltd., Beijing, China) to assess significant differences.

## 5. Conclusions

Over-expression of *VpSBP11* in *Arabidopsis thaliana* was shown to activate the *FUL* gene and, subsequently, the *AP1* and *LFY* genes, all of which are floral meristem identity genes, which caused earlier flowering than in WT plants and changed leaf morphology and the number of transgenic lines.

## Figures and Tables

**Table 1:** The information of primers used in this paper. F indicates forward primer, R indicates reversed primer and the underline indicates restriction sites.

Primer Pairs	Forward and Reverse Primers (5′–3′)	Restriction Enzyme Cutting Site
*VpSBP11*-F1	F:ATGGAAGCTAAGAAGATGGT	none
*VpSBP11*-R1	R:TCATCTGATCTGGAAATGC	none
*VpSBP11*-F2	F:TCCGGCAGCGCTTCTGTCAGC	none
*VpSBP11*-R2	R:TCAGCTGAGTCCTTTCTGCGCCT	none
*AtLFY*-F	F:ACGCCGTCATTTGCTACTCT	none
*AtLFY*-R	R:CTTTCTCCGTCTCTGCTGCT	none
*AtFUL*-F	F:TTGCAAGATCACAACAATTCGCTTCT	none
*AtFUL*-R	R:GAGAGTTTGGTTCCGTCAACGACGAT	none
*AtAP1*-F	F:GAAGGCCATACAGGAGCAAA	none
*AtAP1*-R	R:GGACAACGGAATCTCTCAGC	none
*AtActin1*-F	F:AGGCACCTCTTAACCCTAAAGC	none
*AtActin1*-R	R:ACTGCTCCTGTTGAGCCCTA	none
*VpSBP11*-F3	F:CGCTCTAGAATGGAAGCTAAGAAGATGGTGA	*Xba*I site underlined
*VpSBP11*-R3	R:GGCGGTACCTCTGATCTGGAAATGCTTGTAAG	*Kpn*I site underlined
*VpSBP11*-F4	F:CGTCCCGGGATGGAAGCTAAGAAGATGGT	*Xma*I site underlined
*VpSBP11*-R4	R:GGCGGATCCTCATCTGATCTGGAAATGCTTG	*BamH*I site underlined
Gal4-F	F:GGGCCATGGTAATGAAGCTACTGTCTTCTAT	*Nco*I site underlined
Gal4-R	R:GGGGGATCCTTACTCTTTTTTTGGGTTTG	*Bam*HI site underlined
*VpSBP11*-F5	F:CACGGATCCATGGAAGCTAAGAAGATGGTGA	*Bam*HI site underlined
*VpSBP11*-R5	R:GGCGGTACCTCATCTGATCTGGAAATGCTTG	*Kpn*I site underlined

## Supplementary Materials

Supplementary materials can be found at www.mdpi.com/1422-0067/18/7/1493/s1.

## Abbreviations

SBP	Squamosa promoter binding protein
AP1	Apetala1
FUL	Fruitfull
LFY	Leafy
UTR	Untranslated region
NLS	Nuclear localization signal
WT	Wild-type
