# Supplementary material for: The SBP-Box Gene VpSBP11 from Chinese Wild Vitis Is Involved in Floral Transition and Affects Leaf Development
Source: Int J Mol Sci. 2017 Jul 13;18(7):1493. doi: 10.3390/ijms18071493 (PMC5535983; doi:10.3390/ijms18071493)
Supplement: Supplementary file 1 [file ijms-18-01493-s001.pdf]

Supplementary Files

The SBP-Box Gene *VpSBP11* from Chinese Wild *Vitis* is Involved in Floral Transition and Affects Leaf Development

Hongmin Hou [1,2,3,†], Xiaoxiao Yan [1,2,†],Ting Sha [3],Qin Yan [1,2] and Xiping Wang [1,2,*]

1 State Key Laboratory of Crop Stress Biology in Arid Areas, College of Horticulture, Northwest A&F University, Yangling, Shaanxi 712100, China ; E-Mail: hmhou@qau.edu.cn (H.H.); xiaoxyan@nwafu.edu.cn (X.Y.); yanqin0421@gmail.com (Q.Y.)

2 Key Laboratory of Horticultural Plant Biology and Germplasm Innovation in Northwest China, Ministry of Agriculture, Yangling, Shaanxi 712100, China;

3 College of Horticulture, Qingdao Agricultural University, Qingdao, Shandong 266109, China. shating789000@gmail.com (T.S.)

† These two authors contributed equally to this work

* Correspondence: E-Mail: wangxiping@nwsuaf.edu.cn; Tel.: 86-29-87082429.

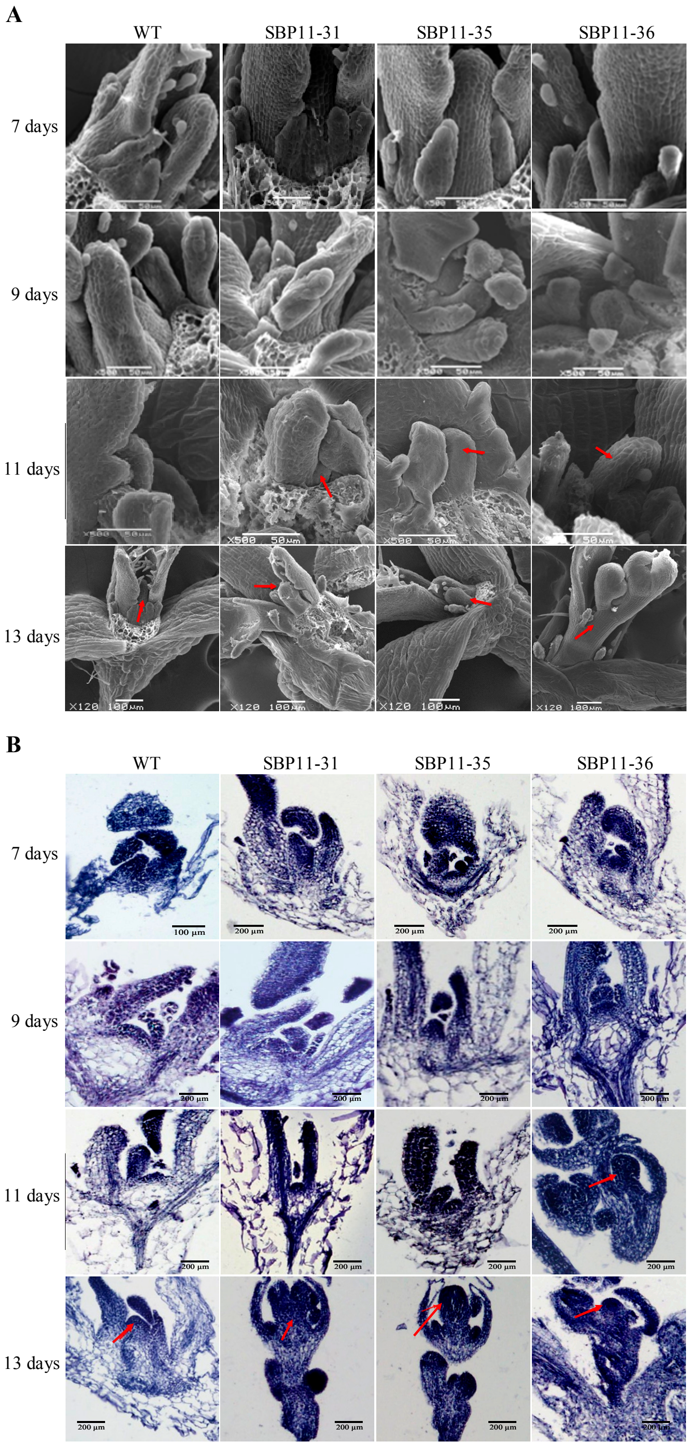

Figure S1. Scanning electron microscope (SEM) analysis (**A**) and paraffin sectioning (**B**) of flower primordia transformations after 7, 9, 11 and 13 days germination in wild-type (WT) and three transgenic lines. The flower primordia at 11 and 13 days after germination are marked by red arrows. The triplicate results of the two methods were consistent.
